# RETRACTION: Evaluation of Perineal Wound Healing and Pain Outcomes after Low‐Angle Mediolateral Episiotomy in Women Undergoing Vaginal Childbirth: A Systematic Review and Meta‐Analysis

**DOI:** 10.1111/iwj.70413

**Published:** 2025-03-21

## Abstract

**RETRACTION:** LuoQ. , LuZ. , and XuB. , “Evaluation of Perineal Wound Healing and Pain Outcomes after Low‐Angle Mediolateral Episiotomy in Women Undergoing Vaginal Childbirth: A Systematic Review and Meta‐Analysis,” International Wound Journal 21, no. 4 (2024): e14826, 10.1111/iwj.14826.38512112 PMC10956536

The above article, published online on 21 March 2024, in Wiley Online Library (http://onlinelibrary.wiley.com/), has been retracted by agreement between the journal Editor in Chief, Professor Keith Harding; and John Wiley & Sons Ltd. Following an investigation by the publisher, all parties have concluded that this article was accepted solely on the basis of a compromised peer review process. The editors have therefore decided to retract the article. The authors did not respond to our notice regarding the retraction.

**RETRACTION:** Q. Luo , Z. Lu , and B. Xu , “Evaluation of Perineal Wound Healing and Pain Outcomes after Low‐Angle Mediolateral Episiotomy in Women Undergoing Vaginal Childbirth: A Systematic Review and Meta‐Analysis,” International Wound Journal 21, no. 4 (2024): e14826, 10.1111/iwj.14826.38512112 PMC10956536

The above article, published online on 21 March 2024, in Wiley Online Library (http://onlinelibrary.wiley.com/), has been retracted by agreement between the journal Editor in Chief, Professor Keith Harding; and John Wiley & Sons Ltd. Following an investigation by the publisher, all parties have concluded that this article was accepted solely on the basis of a compromised peer review process. The editors have therefore decided to retract the article. The authors did not respond to our notice regarding the retraction.

